# Design of the School-Based SI! Program Reintervention Trial for Child Health Promotion

**DOI:** 10.1016/j.jacadv.2026.102748

**Published:** 2026-04-24

**Authors:** Gloria Santos-Beneit, Amaya de Cos-Gandoy, Patricia Bodega, Xavier Orrit, Carla Rodríguez, Mercedes de Miguel, Natalia Montilla, Juan Miguel Fernández-Alvira, Jesús Martínez-Gómez, Raquel Diaz-Muñoz, Domenec Haro, Carles Peyra, Isabel Carvajal, Rodrigo Fernandez-Jimenez, Valentin Fuster

**Affiliations:** a SHE Foundation (Science, Health, and Education), Barcelona, Spain; b Centro Nacional de Investigaciones Cardiovasculares (CNIC), Madrid, Spain; c Hospital Universitario Clínico San Carlos, IdISSC, Madrid, Spain; d Mount Sinai Fuster Heart Hospital, New York, New York, USA; e Centro de Investigación Biomédica En Red en enfermedades CardioVasculares (CIBERCV), Madrid, Spain

**Keywords:** cardiovascular health score, child health promotion, school environment

## Abstract

The SI! Program is a long-term, school-based intervention promoting cardiovascular health across multiple age groups. This article presents the design of a new cluster-randomized controlled trial incorporating a reintervention to strengthen sustainability and promoting a school-family approach. A total of 977 children (age 6.2 years, 51.6% girls) from 50 schools in Spain were recruited. Schools were randomized to a reintervention group (intervention in second grade and reintervention in fifth grade) or a single-intervention group (intervention only in fifth grade). The primary endpoint is the change in the SI!-Child score—diet, physical activity, sleep health, and body mass index—between baseline and 5-year follow-up. The secondary endpoints include 5-year changes in SI!-Child subcomponents, sedentarism, adiposity, and the Life’s Essential 8 score and subcomponents. This revised SI! Program builds on insights from earlier trials, emphasizing adherence and long-term behavior change through a reintervention combined with active engagement of the school community and families. (The SI! Program Reintervention for Elementary Schools Trial [PSIR]; NCT06715358)

## Highlights

• Sustained cardiovascular health from childhood requires long-term, school-based interventions with environmental reinforcement.
• This trial will evaluate a reintervention strategy to enhance adherence and long-term behavior change in children.
• Family and school engagement are key to promoting and maintaining healthy lifestyle habits in youth.
• Future efforts should prioritize scalable, evidence-based interventions with long-term follow-up and community-wide implementation strategies.

The SI! Program (*Salud Integral*–Comprehensive Health) is a multilevel, school-based intervention designed to promote cardiovascular health (CVH) in children and adolescents. Over the past 15 years, the program has been implemented and evaluated across multiple countries and educational stages, with studies assessing different exposures and intervention intensities on a range of CVH parameters.^1^, ^2^, ^3^, ^4^, ^5^, ^6^, ^7^

Evidence from these studies indicates that the retention of knowledge and habits acquired in preschool largely depends on the child’s immediate environment, as children lack the autonomy to make independent lifestyle decisions.^8^ A family-based approach is a critical complement to school-based interventions, as parents largely determine children’s access to food and opportunities for physical activity (PA) during early childhood. Consistently, evidence shows that parental behaviors and early psychosocial environments are strongly associated with children’s long-term cardiometabolic risk and lifestyle patterns later in life.^7^^,^^9^^,^^10^ By early adolescence, dietary habits already require improvement, and levels of PA—especially among girls—begin to decline.^11^^,^^12^ These findings highlight the elementary school years as a particularly favorable period for intervention: children are beginning to make more autonomous choices about their health, yet remain in a stage of high developmental plasticity and are generally free from harmful behaviors such as tobacco or alcohol use.

Throughout the evolution of the SI! Program, several key factors have been identified that influence both its implementation and effectiveness.^7^ Drawing on insights from earlier trials since the program’s launch in 2009, we have now developed a revised version. This updated strategy strengthens the role of the school environment, increases family involvement, and incorporates a reintervention during the elementary school years to promote sustainable, long-term health outcomes.

This trial also introduces the SI!-Child score, a simplified tool adapted from the American Heart Association’s Life’s Essential 8 (LE-8) score.^13^^,^^14^ Focused exclusively on behavioral components, the SI!-Child score offers a practical means of assessing CVH in young populations. The primary aim of the SI! Program Reintervention trial is to assess whether an early exposure to the SI! Program in second grade followed by a reintervention in fifth grade leads to greater improvements in the SI!-Child score than a single intervention delivered only in fifth grade among children aged 7 to 12 years.

## Methods

### Study design, population, and randomization process

The SI! Program Reintervention for Elementary Schools is a cluster-randomized trial, with schools serving as the units of randomization. In Spain, elementary education spans 6 grades and typically includes children aged 6 to 11 years. Eligible schools included public schools and charter schools initially limited to the Central, South, and East administrative areas defined by the Department of Education of the regional government of Madrid (Community of Madrid), as well as the city of Mataró (Barcelona province, Catalonia). To be eligible, schools also had to include at least one first grade class and have an operational canteen.

**Figure 1:**
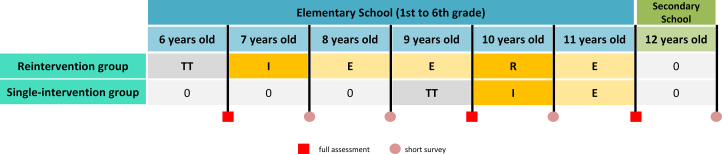
**Study Design of the SI! Program Reintervention Trial** The trial includes 2 intervention groups within the SI! Program Reintervention for Elementary Schools: a reintervention group (with interventions in second and fifth grade) and the single-intervention group (intervention in fifth grade only). In addition to classroom activities, the program incorporates a reinforcement of key messages throughout the school-family environment. Teacher training takes place before intervention delivery. Red squares indicate full assessments; pink circles indicate short surveys E = environment reinforcement (school and home); I = intervention; R = reintervention; TT = teacher training and implementation planification; 0 = no intervention.

All schools meeting the inclusion criteria in the study areas were invited to attend informational meetings. Participation was voluntary at the school level and largely determined by institutional decisions and logistical constraints. Of the 1,026 schools in Madrid and 32 in Mataró that were invited, 50 agreed to participate. One additional school from the West administrative area of Madrid, geographically close to the boundary with the Central area, volunteered to participate and was included because it met all prespecified inclusion criteria. One school subsequently withdrew, resulting in 50 schools included in the study. Participating schools were part of the regular school systems in the study areas and represented diverse socioeconomic contexts, including public and publicly funded private schools. To reach the required sample size (see Sample Size Calculation), recruitment was conducted over 2 consecutive academic years, resulting in 2 enrollment phases: phase I (2023-2024) and phase II (2024-2025). In all, 36 schools from phase I and 15 from phase II agreed to participate (44 from Madrid and 7 from Mataró). Using block randomization stratified by school type (public vs charter), schools were assigned in a 1:1 ratio to either the reintervention group or the single-intervention group. Schools in the reintervention group were allocated to receive the SI! Program from second to sixth grade, with classroom interventions in both second and fifth grades, whereas those in the single-intervention group were allocated to receive the intervention from fifth to sixth grade, with a single classroom intervention in fifth grade (Figure 1). Schools were randomized consecutively in blocks of 6 by an independent researcher, and group allocations were communicated by email.

**Figure 2:**
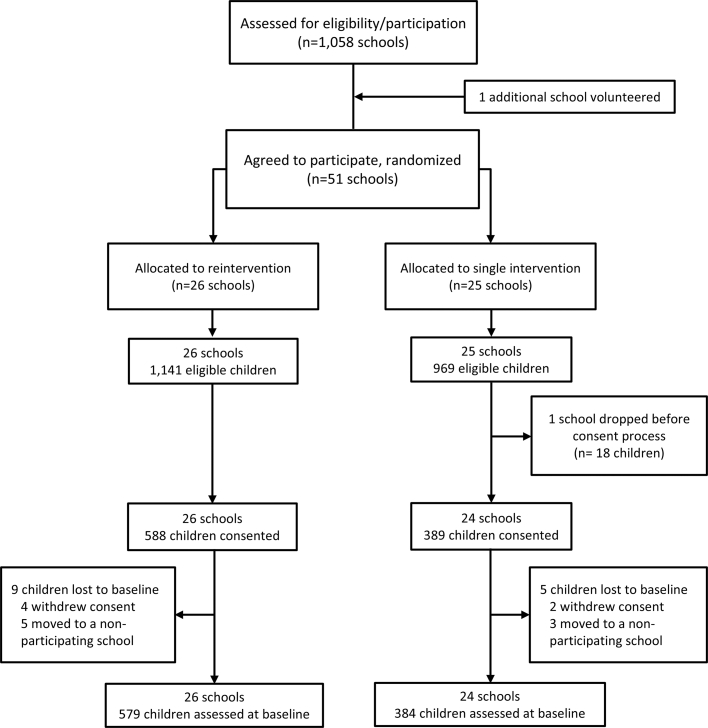
**Study Flowchart of the SI! Program Reintervention Trial** Recruitment was conducted in 2 phases: 36 schools were enrolled in phase I, and 15 in phase II. One school dropped out before baseline assessments, resulting in a total of 50 participating schools. Of these, 26 were randomized to the reintervention group and 24 to the single-intervention group. A total of 977 children provided consent to participate—588 in the reintervention group and 389 in the single-intervention group.

All children in first grade and their families were invited to participate. Enrollment was conditional upon signed informed consent from a parent or legal guardian. Consent forms were distributed via flyers in all first grade classrooms, including a QR code linking to an electronic version of the form, and/or as paper copies collected by teachers on behalf of the research team. In total, 977 children (mean age, 6.2 years; 51.6% girls) from 50 schools were recruited, and 963 were finally enrolled and assessed at baseline. The study flowchart, detailing school and participant recruitment and enrollment, is shown in Figure 2. Participating parents/guardians, children, and school boards have the right to withdraw from the study at any time.

The trial was approved by the Regional Ethics Committee for Research with Medicinal Products (CEIm) of the Community of Madrid (CEIm-R 06/24). All collected data are managed in accordance with Spanish Law 15/1999 on the Protection of Personal Data, ensuring participant confidentiality. The study is registered at ClinicalTrials.gov (NCT06715358). This article reports the study protocol. Although baseline data collection has been completed, no baseline results are presented in this article. Protocol reporting follows the SPIRIT (Standard Protocol Items: Recommendations for Interventional Trials) guidelines,^15^^,^^16^ including the updated checklist (Supplemental Table 1) and the WHO (World Health Organization) Trial Registration Data Set (Supplemental Table 2).

### Intervention

Scientific evidence on health protective factors indicates that effective interventions extend beyond classroom-based activities to engage the entire school as an organizational unit.^17^ These interventions operate at multiple levels, including the broader school community and families.^18^^,^^19^ The underlying attitudes and values embodied by a school—and reflected in the school environment—play a fundamental role in fostering positive, healthy development among students.^20^, ^21^, ^22^ The SI! Program is grounded in the ASE model (Attitude, Social influence, and Efficacy),^23^ which draws on Social Learning Theory (also known as Social Cognitive Theory)^24^ and the Theory of Reasoned Action.^25^

The revised SI! Program retains the original components—diet, PA, body and heart awareness, and emotional management—while introducing a strengthened strategy focused on children’s environments. This enhanced approach aims to foster a sustained culture of health at both school and home and includes a new classroom intervention centered on socioemotional learning (SEL). The curriculum and accompanying educational materials were developed by the SHE Foundation (Science, Health, and Education) using innovative, participatory teaching methods.

The strategy was pilot-tested in 2023 across 4 schools in Madrid, and select resources—such as posters and family activities—were tested in 163 additional schools from the SI! Program cohort across Spain. Teachers implementing the program in their classrooms completed surveys and took part in focus groups to provide feedback on the intervention materials and activities. The findings from this pilot phase informed adaptations to both the curriculum and the evaluation tools prior to the launch of the randomized controlled trial.

### Classroom intervention

The classroom component of the SI! Program consists of teacher-led lessons focused on SEL. These sessions are designed to help children adopt and sustain healthy habits by promoting emotional awareness, self-regulation, self-esteem, and positive social relations. During the intervention period, all teachers have access to the full set of instructional materials, supporting resources, and implementation guidelines via the SI! Program website.

The classroom intervention is scheduled for delivery twice in the reintervention group (second and fifth grade) and once in the single-intervention group (fifth grade only). Each year of implementation consists of 20 hours of instruction divided across 16 sessions, with content tailored to the developmental stages of the target age groups (7-8 years in second grade, and 10-11 years in fifth grade).

### School and family environment

The enhanced strategy for the school environment in the revised SI! Program is designed to encourage self-assessment by the school community and to support the development of feasible plans for improvement. A key tool in this process is the *Healthmeter* (*Saludómetro*), a structured tool that helps schools assess the presence and quality of health-promoting routines and resources (Supplemental Figure 2). The environmental intervention is led by a dedicated *Leading group* composed of school staff, students, and family members committed to health promotion. This group conducts the *Healthmeter* assessment and uses supporting guides—with practical and theoretical examples—to identify and implement appropriate changes. The *Leading group* is also responsible for ensuring the overall implementation of SI! Program activities at the school level.

The strategy also includes an annual school-wide event, *Healthy Day*, which is organized by the school community around a rotating theme drawn from the SI! Program’s core components. The current iteration introduces a new emphasis on rest, broadly defined to include not only sleep routines but also breaks and recovery periods. Activities are tailored to different age groups, and the event is designed to reinforce a shared culture of health across all school levels and among families.

In addition, families receive several newsletters during the academic year. These include instructions for using the *Healthmeter*, provide information about the *Healthy Day* theme, and include other educational resources and guidance to support healthy habits at home.

### Teacher training and implementation monitoring

Formal training is provided annually by the SHE Foundation: 30 hours for the classroom intervention and 20 hours for the school environment component, specifically for members of the *Leading group*. Implementation is monitored through periodic reports submitted by teachers and the *Leading group*, documenting the progress of SI! Program activities at both the classroom and school-environment levels. In addition, the SHE Foundation coordination team issues periodic communications with reminders of key intervention milestones each year, including teacher training activities. Ongoing support is also provided by email and telephone, with the possibility of individual meetings to assist teachers and Leading Groups with any aspect of program implementation. These monitoring procedures will inform the assessment of implementation fidelity, dose, reach, and participant satisfaction, which will be integrated into a composite implementation index.

Additional details on the intervention are provided in the Supplemental Methods.

### Data collection

**Figure 3:**
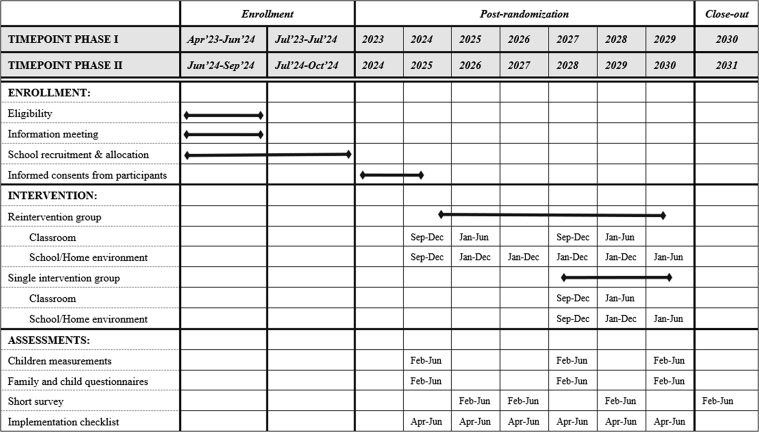
**Study Timeline of the SI! Program Reintervention Trial** Timeline of enrollment, intervention delivery, and assessment activities for schools enrolled in phase I and phase II of the SI! Program Reintervention trial. The figure shows the planned time points for classroom intervention, environment reinforcement (school and home), full health assessments, short survey, and implementation checklists, spanning academic years 2023 to 2031.

The schedule of enrollment, interventions, and assessments for the SI! Program Reintervention trial is summarized in Figure 3. Full participant assessments are scheduled at baseline and at 3- and 5-year follow-up. These include the complete set of LE-8 components, with detailed evaluation both of health behaviors (diet, PA, nicotine exposure, and sleep health) and of health factors (body mass index [BMI], lipid profile, blood glucose, and blood pressure [BP]), as well as body composition indicators such as waist circumference (WC) and body fat mass.

Measurements will be conducted during school hours by a team of trained nutritionists and nurses. Parents/caregivers will complete questionnaires about their child’s and their own lifestyle behaviors through a dedicated data management application, due to the limited ability of young children to accurately report these behaviors. For families without email access, printed copies will be provided.

After each assessment, families will receive a brief medical report summarizing results and including recommendations for follow-up with their primary care physician, if needed. In addition to the main assessments, families will complete short questionnaires at 1-, 2-, 4-, and 6-year follow-up (Figure 1), covering child diet, PA, nicotine exposure, sleep health, and parent/caregiver-reported weight and height. Every effort will be made to follow-up all participants, including those who change schools.

### Diet

Children’s diets will be reported by families using an adapted version of the validated Children's Eating Habits Questionnaire–Food Frequency Questionnaire.^26^, ^27^, ^28^

### Physical activity and sleep

Participants will wear an Actigraph wGT3XBT accelerometer on the nondominant wrist for 7 consecutive days to assess the amount and intensity of PA and sleep patterns.^6^^,^^29^^,^^30^ Families will also report children’s PA using a dedicated questionnaire derived from the validated QAPACE (Quantification de L’Activité Physique en Altitude chez les Enfants) survey.^31^ Additional questions will capture sleep-related habits—including bedtimes and wake-up times on weekdays and weekends and the frequency and duration of naps—and screen time (both leisure and nonleisure) on weekdays and weekends.

### Nicotine exposure

Nicotine exposure will be assessed based on reported use of combustible tobacco or electronic nicotine delivery systems (e-cigarettes or vapes) among participants aged 12 years and older, and/or reported smoking within the immediate household or environment for younger participants.

### Anthropometry and body composition

Body weight will be measured using an OMRON BF511 body composition scale, and height with a Seca 213 portable stadiometer, with participants wearing light clothing and no shoes. Weight and height will be recorded to the nearest 0.1 kg and 0.1 cm, respectively. BMI will be calculated as weight divided by height squared (kg/m^2^).

WC will be measured in triplicate using a Holtain metric tape (accuracy ±0.1 cm), following gentle expiration. The tape will be positioned in a horizontal plane around the abdomen, between the lowest rib and the top of the right iliac crest.

Total body fat will be estimated by bioelectrical impedance analysis with a tetrapolar OMRON BF511 device.

### Glucose and lipid profile

Capillary blood samples will be collected via lancet to measure blood glucose, total cholesterol, high-density lipoprotein cholesterol, low-density lipoprotein cholesterol, non–high-density lipoprotein cholesterol, and triglycerides. Analyses will be performed with a CardioCheck Plus device and PTS Panel test strips.^32^

### Blood pressure

BP will be measured twice at 2 to 3 minutes intervals using an OMRON M6 monitor. If the difference between the 2 systolic readings exceeds 10 mm Hg or the difference between the 2 diastolic readings exceeds 5 mm Hg, a third measurement will be taken. For analysis, the pair of readings with the lowest systolic BP will be selected; in the case of a tie, the pair with the lowest diastolic BP will be used.

### Family

The family questionnaire will collect information on a range of sociodemographic variables, including parental or caregiver country of birth, household income, family structure, parental or caregiver education level, occupation, and social support network. Household income will refer to total net annual income, categorized into quintiles using the most recent regional data from the Spanish National Statistics Institute at baseline.^33^ Family structure will be defined by the adult relatives or caregivers living in the household (eg, biological or adoptive parents, grandparents).^34^ The highest household educational level will be reported, according to the International Standard Classification of Education,^35^ and occupation will be reported according to the European Socioeconomic Classification.^36^ Social support will be assessed with the question “How many persons, other than your family, do you know that you can definitely rely on in cases of need?”^37^

In addition, data will be collected on parental or caregiver lifestyle behaviors, including diet, PA, smoking habits, and self-reported BMI and BP.

### Definition of health scores

#### SI!-Child score

**Figure 4:**
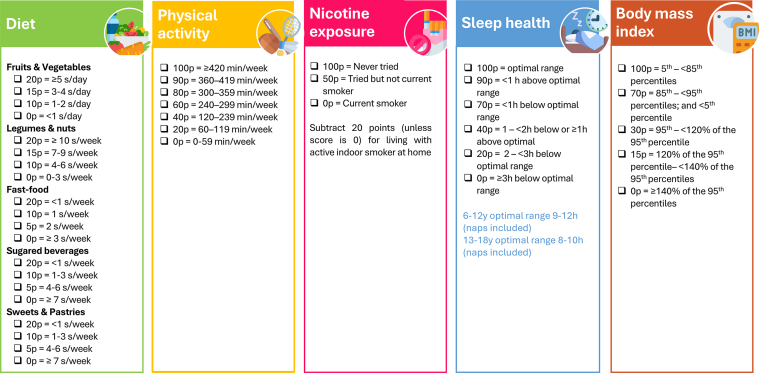
**Components and Scoring Structure of the SI!-Child Score** Each of the 5 individual components of the SI!-Child score is rated on a scale from 0 to 100 points. The overall score is calculated as the unweighted average of the 5 component scores and ranges from 0 to 100. Scores are categorized as follows: poor (0-49), intermediate (50-79), or ideal (80-100). s/day = servings per day; s/week = servings per week.

The SI!-Child score is a simplified tool adapted from the LE-8 scale to assess and monitor lifestyle-related metrics for CVH screening in children and adolescents. This score was designed to capture behavior change and facilitate repeated monitoring in educational school-based interventions. Details of its development and assessments of agreement with the lifestyle-related LE-8 components are provided in the Supplemental Methods and Results. The SI!-Child score incorporates 5 behavioral metrics derived from the LE-8 score: diet, PA, nicotine exposure, sleep health, and BMI. Each metric is scored on a 0 to 100 scale. The overall SI!-Child score is calculated as the unweighted average of the 5 components and categorized as poor (0-49 points), intermediate (50-79 points), or ideal (80-100 points)^13^ (Figure 4). In this trial, nicotine exposure will not be included in the calculation of the SI!-Child score, given the negligible prevalence of active smoking in young children and because nicotine exposure is primarily determined by environmental factors rather than direct child behavior. Therefore, the SI!-Child score will be derived as the unweighted average of the 4 remaining components and categorized according to the established thresholds.

The diet metric is a composite of 5 food groups derived from the food frequency questionnaire: fruits and vegetables, legumes and nuts, fast food, sugared beverages, and sweets and pastries. Each food group is assigned a score from 0 to 20 points.

The PA metric will be assessed primarily through accelerometer-measured weekly minutes of moderate-to-vigorous PA, supplemented by questionnaire responses. A score of 100 is assigned for ≥420 minutes of moderate-to-vigorous PA per week.

Sleep health will be assessed as the average number of hours slept per 24-hour period, using accelerometer data supported by questionnaire information. The ideal range for children aged 6 to 12 years is 9 to 12 hours per day.

BMI categories will be categorized according to age- and sex-adjusted reference percentiles. Normal weight, defined as a BMI between the fifth and 85th percentile, is considered ideal.

#### Life’s Essential 8 score

The LE-8 score of CVH for children and adolescents includes 8 metrics: diet, PA, nicotine exposure, sleep health, BMI, blood lipids, blood glucose, and BP.^13^ The original scoring criteria for these components, along with the specific adaptations used in this study, are summarized in Supplemental Table 3.

#### Hypothesis and endpoints

The study hypothesizes that children exposed to the SI! Program in both second and fifth grade (reintervention group) will show greater improvement in the SI!-Child score and related health parameters over 5 years than those receiving the program only in fifth grade (single-intervention group). The primary endpoint is the between-group difference at 5-year follow-up in the overall SI!-Child score (range, 0-100), encompassing diet, PA, sleep health, and BMI. Secondary endpoints include the between-group differences in the 5-year change in the SI!-Child subcomponents, the LE-8 score and its individual components (diet, PA, nicotine exposure, sleep health, BMI, blood lipids, blood glucose, and BP), as well as measures of sedentary behavior and adiposity markers.

### Statistical methods

#### Sample size calculation

Sample size estimates were based on a cluster-randomized design and calculated using the *clustersampsi* command (Stata version 15.0). Drawing on pilot data (mean SI!-Child score excluding nicotine exposure at ages 7-8 = 70.62; SD = 11.77), a minimum of 40 schools (20 per study arm) was required to detect a 5% difference in SI!-Child score (excluding nicotine exposure) between groups, with 80% power and a 2-sided significance level of 0.05.

Additional parameters included an average of 20 children per school, a coefficient of variation of cluster (school) sizes of 0.51, and an intraclass correlation coefficient of 0.05. To account for potential loss to follow-up, the target sample was increased to 42 schools (21 per arm), corresponding to an expected enrollment of 840 children.

#### Statistical analysis plan

The descriptive analysis will summarize baseline data, including demographic variables (eg, gender, age, place of birth), socioeconomic indicators (eg, household income, parental or caregiver education level and country of birth), and study-specific variables (eg, SI!-Child scores; lifestyle habits such as diet, sleep, and PA; adiposity measures; and blood parameters). Continuous variables will be presented as means and SDs, and categorical variables as frequencies and percentages.

Primary endpoint differences will be evaluated using all available measurements at baseline and at 5-year follow-up. The intervention effect will be estimated using multilevel mixed-effects models for repeated measures. The model will include fixed effects for time of assessment (baseline vs follow-up), randomization group (reintervention vs single-intervention), and the interaction between time and randomization group. The treatment effect will be estimated from the time-by-randomization interaction term, representing the between-group difference in change in SI!-Child score over time. Random intercepts for school and participant will be included to account for clustering at the school level and repeated measurements within individuals. Similar multilevel mixed-effects logistic regression models will be applied for categorical outcomes, and the same analytical strategy will be used to evaluate secondary endpoints.

The primary analysis will follow the intention-to-treat principle, including all randomized enrolled participants according to their allocated group. Models will be estimated using maximum likelihood, which provides valid inference under a missing-at-random assumption and uses all available data without requiring imputation. Models may additionally adjust for prespecified baseline covariates (eg, baseline outcome value, age, gender, and stratification variable) to improve precision.

Sensitivity analyses will be conducted to evaluate the robustness of the findings to alternative assumptions about missing data. These will include 1) complete-case analyses restricted to participants with both baseline and follow-up outcome measurements; 2) multiple imputation procedures; 3) additional sensitivity analyses exploring potential missing-not-at-random mechanisms, and other statistical approaches such as cluster-level analyses or Analysis of Covariance models of follow-up outcomes adjusted for baseline values.

The SI! Program Reintervention design permits the simultaneous testing of multiple hypotheses. For each research objective, a specific statistical analysis plan will be developed. At each assessment time point, cross-sectional associations between independent variables and outcomes of interest will be evaluated using multivariate linear regression models for continuous variables and logistic regression models for categorical variables. Covariate adjustment will be defined a priori based on the specific research question and determined through a combination of clinical relevance and statistical criteria.

All statistical analyses will be conducted using Stata 15.0 or higher (StataCorp) or R software.

## Discussion

Over the past 50 years, widespread lifestyle changes and an increase in cardiovascular risk factors—such as obesity, hypertension, dyslipidemia, and diabetes mellitus—have contributed to a global rise in the incidence and prevalence of cardiovascular events, including heart failure and stroke.^13^^,^^38^ At the same time, preventive strategies promoting healthy lifestyles have helped reduce cardiovascular mortality rates.^13^^,^^38^ Despite these gains, determining when and how to intervene to preserve CVH from early childhood remains a persistent challenge.

The SHE Foundation, in collaboration with CNIC (*Centro Nacional de Investigaciones Cardiovasculares*, Spanish National Centre for Cardiovascular Research), has developed and evaluated multiple health promotion programs that use education as a tool for cultivating healthy habits from childhood through adulthood, to foster a culture in which people of all ages prioritize health and well-being.^7^^,^^39^ Similar programs targeting children and adolescents exist worldwide.^40^ While childhood is widely recognized as a critical period for establishing lifelong health behaviors,^41^ it is not sufficient to address a single cardiovascular risk factor—such as obesity—or to focus on a single population subgroup.^42^ Instead, a comprehensive approach is needed that integrates emotional, behavioral, and environmental dimensions across the broader community.^7^^,^^41^^,^^43^ The life-course approach provides a compelling framework for this purpose: supporting health and its social determinants across all life stages increases healthy life expectancy and generates significant individual, societal, and economic benefits.^43^

Primordial prevention strategies aim to halt the emergence of risk factors before they lead to clinical disease. In this context, urban provisioning systems—encompassing food, water, energy, transportation, buildings, and sanitation—have been extensively studied and are increasingly integrated into public health and sustainability planning.^44^ However, population-wide education, particularly at early stages of life, remains underemphasized in many of the frameworks and reports guiding these efforts.^43^^,^^44^

The Transtheoretical Model of Health Behavior Change outlines 6 stages of individual change: precontemplation, contemplation, preparation, action, maintenance, and termination.^45^ According to this model, improvements in health literacy can initiate progress through these stages—beginning with changes in knowledge (precontemplation and contemplation), followed by shifts in attitudes (preparation), and ultimately producing sustained behavior change (action, maintenance, and termination).^7^ In this light, education becomes a cornerstone of preventive health strategies, offering a low-cost, scalable alternative to more invasive interventions.

To fully realize the benefits of prevention, scientific knowledge must be translated into practical, community-level action—beginning at the household level and extending to the neighborhood. This includes fostering healthy diets, expanding opportunities for PA, supporting effective emotional regulation, and promoting smoking cessation. For this to succeed, science must be embedded in society, and society must, in turn, be empowered to act on science.

In the upcoming trial of the revised SI! Program (Central Illustration), the whole-school strategy underscores the importance of collective action in promoting meaningful and sustained health behavior change.^46^^,^^47^ By engaging multiple stakeholders—students, educators, school staff, and families—particularly through the *Leading group* and the *Healthmeter* tool, the program fosters a shared sense of ownership and responsibility for health-related goals.

**Central Illustration:**
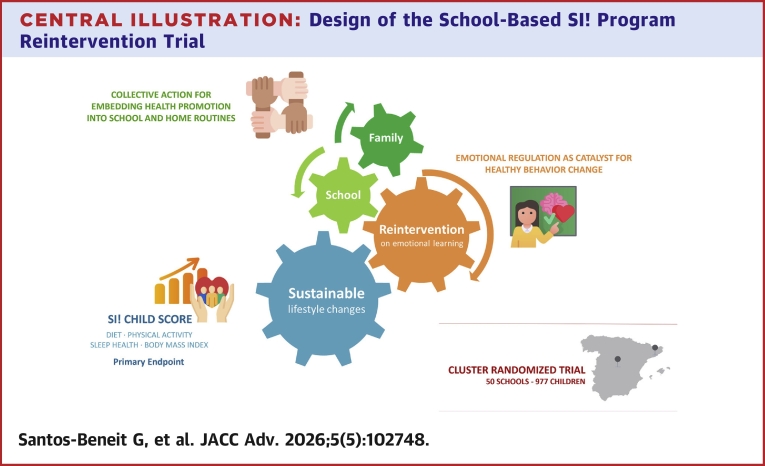
**Design of the School-Based SI! Program Reintervention Trial** This cluster-randomized controlled trial enrolled 977 children from 50 public and charter schools in Madrid and Barcelona, Spain. Schools were randomized to one of 2 arms: a reintervention group (first intervention in second grade and reintervention in fifth grade) or a single-intervention group (intervention only in fifth grade). This revised SI! Program builds on insights from earlier trials, emphasizing adherence and long-term behavior change through a reintervention strategy on socioemotional learning combined with active engagement of the school community and families. The goal is to strengthen the program's impact—assessed by the SI! Child lifestyle score—and support the adoption of lasting healthy habits, thereby contributing to the reduction of cardiovascular disease burden.

This collective approach not only distributes workload and accountability but also strengthens motivation and commitment across the school community. Within this model, collective action serves as a catalyst for reinforcing social norms around health, encouraging peer support, and embedding health-promoting practices into the rhythms of daily school life through resources such as posters, stickers, and structured daily routines.

Equally important is the program’s emphasis on participatory processes, which enable members of the school community to shape actions according to their needs, values, and local context. This approach aims to increase the relevance and acceptability of activities, while also helping to shift power dynamics by elevating the voices of students and less formally recognized contributors, such as support staff and parents/caregivers. This inclusive model of engagement has the potential to foster a platform for continuous feedback and adaptation. Together with the program’s reintervention strategy, this emphasis on inclusion may represent a key mechanism for achieving long-term impact.

The revised design aligns implementation timing with teachers’ capacity to deliver the curriculum effectively, supporting adherence at levels associated with the greatest impact—defined as completion of more than 75% of program content.^2^^,^^3^ Beyond the collective intervention strategy, the strengthened focus on SEL—specifically targeting emotional regulation—adds an innovative dimension that may further enhance effectiveness, given its strong links to the adoption and sustained practice of healthy behaviors.^48^^,^^49^ The active involvement of the entire school community in periodic reinterventions that incorporate SEL may be essential for embedding health promotion into school routines and maximizing long-term impact.

One of the key innovations in the revised SI! Program is the introduction of the SI!-Child score, adapted from the American Heart Association’s recently proposed LE-8^50^ which includes clinical metrics that require blood sampling. However, because clinical measures often remain unaltered over the short term, such composite scores may be less suitable for detecting the effects of primordial prevention strategies in young populations.^6^ The SI!-Child score was designed as a simple, reliable, and easy-to-use tool for assessing and tracking CVH in children and adolescents.

Other screening tools that focus on lifestyle and other risk factors while excluding laboratory measures have proved to be reliable instruments for the assessment of CVH in adults.^51^^,^^52^ The Fuster BEWAT score (BP, Exercise, [body] Weight, diet [Alimentation], and Tobacco use) has been found to perform similarly to the Life’s Simple 7 score in terms of its ability to predict subclinical atherosclerosis, left ventricular abnormalities, and target organ damage.^53^, ^54^, ^55^ Such screening tools do not require specialized instruments or direct measurements, can be easily self-reported, and are particularly valuable in low- and middle-income countries where simpler and more comprehensive assessment methods are needed.^56^

The selection of appropriate evaluation tools is critical in any intervention trial. The integration of gold standard social science methods with minimally invasive biophysiological data collection represents a promising frontier in health promotion research.^13^^,^^57^ However, tools must be carefully tailored to participants’ age, cognitive development, and attention span.^58^ In early childhood, moreover, direct health indicators often show little change over short- to mid-term follow-up, since conditions such as dyslipidemia and hypertension are relatively uncommon in this population.^59^^,^^60^

This was evident in the SI! Program for Secondary Schools trial, where significant improvements in BP and cholesterol were difficult to detect, and behaviors such as smoking were uncommon (92.3% [95% CI: 90.7-93.6] of 12-year-olds reported never having smoked).^6^ Assessment tools must therefore be closely aligned with the research question and capable of detecting meaningful, age-appropriate changes, even if this means excluding other potentially valuable measures. For example, SI! Program trials with preschoolers^7^ used relatively simple KAH (Knowledge, Attitudes, and Habits) questionnaires, which may lack sensitivity when applied across broad age ranges that span considerable variation in cognitive development. Consequently, the trials in preadolescents^2^ and adolescents^6^ employed more complex instruments, such as those assessing PA^31^ and dietary intake via food frequency questionnaires.^26^^,^^61^

A good example comes from the SI! Program for Elementary Schools trial (1,770 children aged 6-11), which tested different intervention intensities across grades: content was either distributed evenly across the 6 grades (6-11 years), concentrated in the first 3 grades (6-8 years), or concentrated in the final 3 grades (9-11 years), with increased instructional hours allocated to the later years of Elementary School.^5^ While all the intervened groups showed significant improvements in several adiposity indicators, no changes were detected in KAH score. The *Lancet Diabetes & Endocrinology Commission* recently recommended confirming BMI estimates of excess adiposity through direct body fat measurement or additional anthropometric criteria—such as WC, waist-to-hip ratio, or waist-to-height ratio—using validated age- and gender-appropriate methods and cutoffs.^62^ In line with this recommendation, interventions should incorporate a broader set of indicators that more precisely reflect adiposity distribution and may capture differences not seen with traditional obesity measures, especially in this age range.^63^, ^64^, ^65^

Previous SI! Program trials found no between-group differences in BMI,^1^^,^^3^^,^^4^ but did detect favorable intervention effects on subscapular skinfold thickness in preschoolers^4^ and on WC and waist-to-height ratio in elementary school children.^5^ In the Elementary Schools trial, children in the intervention groups who began the program at age 6 showed significant improvements in adiposity markers after 6 years.^5^ Nevertheless, BMI was retained in the SI!-Child score, in line with American Heart Association recommendations for the LE-8.

Finally, the use of a CVH score derived from the LE-8 framework enables robust comparability across studies, thereby broadening the scope and applicability of the trial’s findings. At the same time, the exclusion of blood-based metrics enhances the SI!-Child score’s sensitivity to short- and mid-term changes in lifestyle-related behaviors—aligning with the SI! Program’s goal of promoting behavior change through periodic reintervention. Clinical indicators often remain within the ideal range throughout childhood and adolescence, potentially masking early declines in lifestyle-related CVH.^6^^,^^66^, ^67^, ^68^ Screening tools like the SI!-Child score are therefore essential for monitoring overall CVH and evaluating the effectiveness of health promotion programs in young populations.

## Conclusions

The revised design of the SI! Program underscores the central role of collective action and periodic reintervention in driving sustainable behavior change within the school community. By actively engaging students, teachers, families, and staff, the revised SI! Program fosters a shared sense of ownership, cultural relevance, and commitment to health-promoting values. This participatory approach enhances responsiveness to local needs and strengthens community engagement, thereby increasing the overall effectiveness of health promotion efforts in both schools and households.

Building on more than 15 years of experience across multiple SI! Program trials—and informed by established models of health promotion and implementation science—the revised design offers a promising framework for understanding the implementation dynamics of participatory interventions with repeated exposures. The updated SI! Program approach highlights the potential of collective engagement to shape long-term health behaviors and, ultimately, improve CVH outcomes in children and adolescents.

## Funding support and author disclosures

The SI! Program Reintervention for Elementary Schools trial is funded by the “la Caixa” Foundation (grant number LCF/PR/CE16/10700001) and the SHE Foundation and is supported by the Fundación Nemesio Diez. This trial is supported by the Department of Health of the Government of Catalonia (Department de Salut de la Generalitat de Catalunya) under grant [SLD168/25/000001]. Dr Martínez-Gómez is funded by contract FPU21/04891 (Ayudas para la formación de profesorado universitario, FPU-2021) from the Ministerio de Ciencia, Innovación y Universidades (MCIU). Dr Diaz-Muñoz is funded by Sara Borrell postdoctoral contract CD24/00084 from the Instituto de Salud Carlos III (ISCIII) with co-funding from the European Union (EU). Dr Fernandez-Jimenez is supported by project PI22/01560, which is also funded by the ISCIII with EU co-funding. The CNIC is supported by the ISCIII, the MCIU, and the Pro CNIC Foundation and is a Severo Ochoa Center of Excellence (grant CEX2020-001041-S, funded by MCIN/AEI/10.13039/ 501100011033). The authors have reported that they have no relationships relevant to the contents of this paper to disclose.

## Supplementary material

Supplemental Materail
