# Supplementary material for: Design of the School-Based SI! Program Reintervention Trial for Child Health Promotion
Source: JACC Adv. 2026 Apr 24;5(5):102748. doi: 10.1016/j.jacadv.2026.102748 (PMC13129425; doi:10.1016/j.jacadv.2026.102748)
Supplement: Supplemental Materail [file mmc1.docx]

SUPPLEMENTAL MATERIALS

[SUPPLEMENTAL METHODS 2](#_Toc224221354)

[Additional information for SPIRIT guidelines 2](#_Toc224221355)

[Detailed Description of the SI! Program Intervention 4](#_Toc224221356)

[Classroom materials 4](#_Toc224221357)

[School and family environment materials 4](#_Toc224221358)

[Development of the SI! Child score 6](#_Toc224221359)

[Participants and study design 6](#_Toc224221360)

[The Life’s Essential 8 score 6](#_Toc224221361)

[The SI!-Child score 6](#_Toc224221362)

[Scoring points 8](#_Toc224221363)

[Data collection 8](#_Toc224221364)

[Statistical analysis 9](#_Toc224221365)

[SUPPLEMENTAL RESULTS 10](#_Toc224221366)

[SUPPLEMENTAL TABLES 11](#_Toc224221367)

[Supplemental Table 1. 11](#_Toc224221368)

[Supplemental Table 2. 16](#_Toc224221369)

[Supplemental Table 3. 18](#_Toc224221370)

[Supplemental Table 4. 21](#_Toc224221371)

[SUPPLEMENTAL FIGURES 22](#_Toc224221372)

[Supplemental Figure 1 22](#_Toc224221373)

[Supplemental Figure 2. 23](#_Toc224221374)

[Supplemental Figure 3. 24](#_Toc224221375)

[Supplemental Figure 4. 25](#_Toc224221376)

[Supplemental Figure 5 26](#_Toc224221377)

[SUPPLEMENTAL REFERENCES 27](#_Toc224221378)

# SUPPLEMENTAL METHODS

## Additional information for SPIRIT guidelines

Data management, data confidentiality and access, and data monitoring

Data from enrolled children participants, and families are collected on electronic forms, with paper forms available for families with limited internet access. Data collected on paper forms will be kept behind two locked doors. Electronic data will be stored in password-protected files on the local server at the SHE Foundation. Access to paper and electronic data will be restricted to the scientific team: RF-J, AdC-G, JMF-A, JM-G, and GS will have access to the full trial dataset. Additional statistical experts may be consulted in the process of data cleaning and analysis, and will have access only to de-identified data.

Because this is a minimal risk study, there will be no data monitoring committee.

Interim analysis and stopping guidelines

Because this is a minimal risk study, there will no interim analysis with the purpose of taking decisions to continue or terminate the trial.

Management of adverse events and other unintended trial effects

The SHE Foundation collects and reports any adverse incidents during the intervention and data collection process. Other measures are considered unnecessary, as the trial is a minimal risk study.

Research ethics approval and protocol amendments

In the event of protocol modifications, a letter with notification about the changes will be submitted to the Regional Ethics Committee for Research with Medicinal Products (CEIm) of the Community of Madrid (CEIm-R), Spain. Amendments to the protocol will be tracked and dated, and updated on clinicaltrials.gov. Routine annual reports will be submitted to inform the Ethics Committees of the progress of the research plan and goals.

Dissemination policy

The study results will be published in international peer-reviewed journals and presented at major international meetings. The main study results will be communicated to the specialized and general media, including social media, by means of ad hoc press releases and online notices. This dissemination activity will be supported by dedicated communication departments at the researchers’ affiliated institutions.

Authorship eligibility guidelines and author contributions

We follow the International Committee of Medical Journal Editors (ICMJE) recommendations for authorship criteria. VF conceived the overall study. GS-B, PB, MdM, AdC-G, IC, CR, DH, XO, and RF-J made substantial conceptual contributions to the study design. GS-B, PB, MdM, AdC-G, DH, NM, XO, CR, and RD-M coordinated school and participant recruitment, the consent process, and/or data collection. CR, XO, MdM, DH, and IC coordinated the development and implementation of the educational program. AdC-G managed the project database and led the statistical analysis. RF-J, JMF-A, and JM-G assisted with data analysis and contributed to the statistical analysis plan**.** CP led project administration and funding acquisition. GS-B drafted the first version of the manuscript together with AdC-G and RF-J. All authors critically reviewed the manuscript for intellectual content and approved the final version for publication. The trial sponsor and funders had no role in the design, conduct, analysis, or reporting of the trial.

## Detailed Description of the SI! Program Intervention

### Classroom materials

For the classroom component, 16 SEL sessions are scheduled to develop the socio-emotional skills required for the internalization and maintenance of healthy habits. The curriculum addresses socio-emotional competencies through six basic emotions—pride, love, fear, sadness, anger, and joy—and explores how these emotions influence children’s decisions and, consequently, their behaviors and habits.

Each session is designed to be delivered in approximately 45 minutes and includes teaching resources, activity sheets, and games that apply different pedagogical strategies depending on the learning objective. These resources include: (1) a session outline and a teaching guide for teachers to facilitate discussion, reflection, and guided conversation through structured questions; (2) the narrative series of Hugo’s Dilemmas, consisting of 16 chapters (one per session) used to introduce the topic, generate interest, and activate students’ prior knowledge and experiences; (3) experiential games designed to engage the bodily dimension and allow students to experience emotions and learning in an embodied way; and (4) reflective, simple writing, and artistic activities that help students represent and consolidate the key messages of the session.

### School and family environment materials

For the school and family environment components, the SI! Program provides a wide range of educational resources, including posters, infographics, stickers, and other materials supporting health promotion. Both teachers and families have access to these materials through the study intranet (password-protected) and through the Healthmeter platform. In addition, participating schools receive a welcome pack containing printed versions of selected resources (**Supplemental Figure 1**). All materials are organized according to the core components of the SI! Program: active living, rest, nutrition, emotional well-being, and comprehensive health (*Salud Integral*, SI!).

The Healthmeter is designed as a participatory tool that allows the entire school community—including teachers, non-teaching staff, students, and families—to contribute their perspectives. Its goal is to promote awareness of existing habits both at school and at home and to assess whether these practices align with health-promoting behaviors. This interactive resource is connected to all educational materials available through the SI! Program intranet, enabling both schools and families to access information and guidance to help identify and implement improvements in school and household routines. A child-friendly version has also been developed, featuring shorter questions and answers adapted to younger ages and including audio narration to facilitate accessibility. During navigation, the system asks questions related to the different components of the program and provides multiple response options. Based on the selected responses, the platform proposes specific challenges aimed at improving the assessed area, along with an explanation of the associated health benefits and a recommended resource to support implementation of the suggested change (**Supplemental Figure 2**).

## Development of the SI! Child score

### Participants and study design

The SI!-Child score was developed using longitudinal data from the SI! Program for Secondary Schools trial. This cluster-randomized trial was designed to evaluate the effects of two multicomponent educational health promotion strategies—of differing duration and intensity—on the cardiovascular health (CVH) of adolescents aged 12 and 16 years (Clinical Trials number: NTC03504059)^1^. The trial enrolled 1326 participants from 24 schools located in Barcelona and Madrid, Spain. The study was approved by the corresponding Committees for Ethical Research, and all participants provided written consent. Data were collected and handled in accordance with Spanish Law 15/1999 regarding the Protection of Personal Data. Further details of the trial design and primary results have been published elsewhere^2^.

### The Life’s Essential 8 score

The Life’s Essential 8 (LE-8) score for CVH in children and adolescents includes eight metrics: diet, physical activity (PA), nicotine exposure, sleep health, body mass index (BMI), blood lipids, blood glucose, and blood pressure^3^.

### The SI!-Child score

The SI!-Child score adapts the five lifestyle-related metrics from the LE-8 score: diet, PA, nicotine exposure, sleep health, and BMI.

The diet metric is reconfigured as a composite of five food groups: fruits and vegetables, legumes and nuts, fast food, sugared beverages, and sweets and pastries. Each food group is given a score ranging from 0 to 20 points. For the LE-8 score, the American Heart Association proposes the use of one of two dietary questionnaires that adhere to specific eating patterns for the USA population: the Dietary Approaches to Stop Hypertension and the Mediterranean Eating Pattern for Americans^3-5^. In contrast, the SI!-Child score proposes the use of dietary indicators, widely used as proxies of diet quality and easily derived from diverse tools such as food frequency questionnaires and diet quality questionnaires^6^. Moreover, the use of the selected food groups considers overall healthy dietary habits rather than following a specific type of diet, so it is also applicable to dietary restrictions such as vegetarianism or halal. Thus, while the diet component of the LE-8 focuses on the USA population, the SI!-Child is suitable for diverse traditional dietary patterns. Furthermore, dietary indicators were used in the Life’s Simple 7, but without a scoring range for each dietary indicator: responses for each dietary indicator were assigned a score of 1 point if the recommendation was met or 0 points if it was not. This scoring system thus lacked sensitivity to detect changes or improvements in diet that fall short of the recommendation, reducing the chance of finding differences derived from educational programs. To overcome this limitation, the SI!-Child proposes a range for each of the dietary indicators, allowing a wider scoring spectrum for the overall metric, which is especially important in the context of health promotion.

The PA metric follows the LE-8 scoring framework but introduces a stricter lower threshold for the least active categories: participants performing fewer than 60 minutes of moderate-to-vigorous PA (MVPA) per week receive 0 points, while those reporting 60 to 119 minutes per week receive 20 points. In contrast, the LE-8 awards 20 points for 1 to 119 minutes of MVPA per week. Therefore, LE-8 would assign 20 points to adolescents doing less PA than stipulated by the standard school curriculum^7^. While this difference makes the SI!-Child PA metric more restrictive, it also makes it more sensitive to detect subtle changes in the lowest PA levels, in line with the main goal of health promotion interventions.

For nicotine exposure, the SI!-Child score uses the LE-8 cut-off criteria but does not differentiate between combustible tobacco and inhaled nicotine-delivery systems due to the non-availability of this information.

Sleep health is scored according to LE-8 thresholds, with ideal sleep defined as 9–12 hours/day for children aged 6–12 and 8–10 hours/day for adolescents aged 13–18. However, in the current analysis, the 8–10 hour criterion was applied to all participants, as they were enrolled in the same secondary education grade at baseline.

BMI scoring also follows the LE-8 system, except that participants with BMI below the 5th percentile are assigned 70 points. In the LE-8, scoring in this category depends on clinical judgment in each individual case. Given that the SI!-Child is intended for use for and by the general population, especially in health promotion interventions for which clinicians may not be available, it is easier to score the BMI component according to previously defined categories.

### Scoring points

For both the LE-8 and SI!-Child scores, each individual metric is scored on a 0–100 scale. The overall score is calculated as the unweighted average of the component scores (range, 0–100 ) and is categorized as poor (0–49), intermediate (50–79), or ideal (80–100)^3^. The specific scoring criteria used to generate the LE-8 and SI!-Child scores in this analysis are summarized in **Supplemental Table 3**.

### Data collection

Longitudinal data were collected through questionnaires and direct measurements at three time points: baseline (September–December 2017), 2-year follow-up (February–June 2019), and 4-year follow-up (February-June 2021). At each time point, the analysis included only adolescents with complete data for all LE-8 and SI!-Child metrics. Additional details on data collection procedures are available elsewhere^1,2^.

### Statistical analysis

Differences in changes over time in the overall SI!-Child and LE-8 scores were assessed using unpaired Student t-tests. Equality of variance was evaluated with F-tests, with corrections applied when variance was unequal. Categorization of CVH as poor, intermediate, or ideal based on LE-8 and SI!-Child scores was visualized using Sankey diagrams. Bland–Altman plots were used to assess agreement between the lifestyle-related metrics of the LE-8 score (diet, PA, nicotine exposure, sleep health, and BMI) and both the overall and individual metric SI!-Child scores. Statistical significance was defined as P <.05. All analyses were performed using Stata version 15 (StataCorp, College Station, Texas).

# SUPPLEMENTAL RESULTS

LE-8 and SI!-Child scores were calculated for 796 adolescents at baseline, 760 at 2-year follow-up, and 683 at 4-year follow-up. Both scores decreased over time, but the decrease was significantly greater for the SI!-Child score. The between-score difference was –2.45 points at 2-year follow-up (95% CI: –3.00 to –1.90) and –5.19 points at 4-year follow-up (95% CI: –5.80 to –4.58) (**Supplemental Table 4**).

At baseline, the SI!-Child score classified fewer adolescents than the LE-8 in the ideal CVH category (32.7% vs 44.1%) and more adolescents in the poor CVH category (1.0% vs. 0.3%). Both scores classified most adolescents as having intermediate CVH: 443 (55.7%) using the LE-8 score and 528 (66.3%) using the SI!-Child score. Similar trends were observed at 2-year and 4-year follow-up: the proportion of participants with ideal CVH decreased over time, while the proportion with poor CVH increased, regardless of the scoring method used **(Supplemental Figure 3)**.

Bland–Altman plots showed good agreement between the SI!-Child score and the lifestyle-related metrics of the LE-8 score. Mean biases at baseline, 2-year follow-up, and 4-year follow-up were 0.77 (95% limits of agreement: –4.60 to 6.13), 1.08 (–4.58 to 6.73), and 0.96 (–4.92 to 6.85) points, respectively **(Supplemental Figure 4)**. Among the lifestyle metrics, the largest difference between the two scores was recorded for diet **(Supplemental Figure 5)**.

# SUPPLEMENTAL TABLES

Supplemental Table 1. **SPIRIT 2025 checklist of items to address in a randomized trial protocol**

| **Section / Topic** | **No** | **SPIRIT 2025 checklist item description** | **Reported on page no.** |
| --- | --- | --- | --- |
| **Administrative information** | | |  |
| Title and structured summary | 1a | Title stating the trial design, population, and interventions, with identification as a protocol | 1 |
|  | 1b | Structured summary of trial design and methods, including items from the World Health Organization Trial Registration Data Set | 1 |
| Protocol version | 2 | Version date and identifier | 1, 3, S16 |
| Roles and responsibilities | 3a | Names, affiliations, and roles of protocol contributors | 1 |
|  | 3b | Name and contact information for the trial sponsor | S16 |
|  | 3c | Role of trial sponsor and funders in design, conduct, analysis, and reporting of trial; including any authority over these activities | S3 |
|  | 3d | Composition, roles, and responsibilities of the coordinating site, steering committee, endpoint adjudication committee, data management team, and other individuals or groups overseeing the trial, if applicable | S3 |
| **Open science** | | |  |
| Trial registration | 4 | Name of trial registry, identifying number (with URL), and date of registration. If not yet registered, name of intended registry | 1, 3, S16 |
| Protocol and statistical analysis plan | 5 | Where the trial protocol and statistical analysis plan can be accessed | S2 |
| Data sharing | 6 | Where and how the individual de-identified participant data (including data dictionary), statistical code, and any other materials will be accessible | S2 |
| Funding and conflicts of interest | 7a | Sources of funding and other support (e.g., supply of drugs) | 12, S16 |
|  | 7b | Financial and other conflicts of interest for principal investigators and steering committee members | 12 |
| Dissemination policy | 8 | Plans to communicate trial results to participants, healthcare professionals, the public, and other relevant groups (e.g., reporting in trial registry, plain language summary, publication) | S3 |
| **Introduction** | | |  |
| Background and rationale | 9a | Scientific background and rationale, including summary of relevant studies (published and unpublished) examining benefits and harms for each intervention | 1, 2 |
|  | 9b | Explanation for choice of comparator | 2, 3 |
| Objectives | 10 | Specific objectives related to benefits and harms | 2 |
| **Methods: Patient and public involvement, trial design** | | |  |
| Patient and public involvement | 11 | Details of, or plans for, patient or public involvement in the design, conduct, and reporting of the trial | N/A |
| Trial design | 12 | Description of trial design including type of trial (e.g., parallel group, crossover), allocation ratio, and framework (e.g., superiority, equivalence, non-inferiority, exploratory) | 2, 3 |
| **Methods: Participants, interventions, and outcomes** | | |  |
| Trial setting | 13 | Settings (e.g., community, hospital) and locations (e.g., countries, sites) where the trial will be conducted | 2 |
| Eligibility criteria | 14a | Eligibility criteria for participants | 2 |
|  | 14b | If applicable, eligibility criteria for sites and for individuals who will deliver the interventions (e.g., surgeons, physiotherapists) | 2 |
| Intervention and comparator | 15a | Intervention and comparator with sufficient details to allow replication including how, when, and by whom they will be administered. If relevant, where additional materials describing the intervention and comparator (e.g., intervention manual) can be accessed | 3-5, S4, S5 |
|  | 15b | Criteria for discontinuing or modifying allocated intervention/comparator for a trial participant (e.g., drug dose change in response to harms, participant request, or improving/worsening disease) | N/A |
|  | 15c | Strategies to improve adherence to intervention/comparator protocols, if applicable, and any procedures for monitoring adherence (e.g., drug tablet return, sessions attended) | 5, Figure 3 |
|  | 15d | Concomitant care that is permitted or prohibited during the trial | N/A |
| Outcomes | 16 | Primary and secondary outcomes, including the specific measurement variable (e.g., systolic blood pressure), analysis metric (e.g., change from baseline, final value, time to event), method of aggregation (e.g., median, proportion), and time point for each outcome | 8 |
| Harms | 17 | How harms are defined and will be assessed (e.g., systematically, non-systematically) | N/A |
| Participant timeline | 18 | Time schedule of enrollment, interventions (including any run-ins and washouts), assessments, and visits for participants. A schematic diagram is highly recommended (see Figure) | Figure 3 |
| Sample size | 19 | How sample size was determined, including all assumptions supporting the sample size calculation | 8 |
| Recruitment | 20 | Strategies for achieving adequate participant enrollment to reach target sample size | 2, 3 |
| **Methods: Assignment of interventions** | | |  |
| Randomization: |  |  |  |
| Sequence generation | 21a | Who will generate the random allocation sequence and the method used | 3 |
|  | 21b | Type of randomization (simple or restricted) and details of any factors for stratification. To reduce predictability of a random sequence, other details of any planned restriction (e.g., blocking) should be provided in a separate document that is unavailable to those who enroll participants or assign interventions | 2, 3 |
| Allocation concealment  mechanism | 22 | Mechanism used to implement the random allocation sequence (e.g., central computer/telephone; sequentially numbered, opaque, sealed containers), describing any steps to conceal the sequence until interventions are assigned | 2, 3 |
| Implementation | 23 | Whether the personnel who will enroll and those who will assign participants to the interventions will have access to the random allocation sequence | 3 |
| Blinding | 24a | Who will be blinded after assignment to interventions (e.g., participants, care providers, outcome assessors, data analysts) | N/A |
|  | 24b | If blinded, how blinding will be achieved and description of the similarity of interventions | N/A |
|  | 24c | If blinded, circumstances under which unblinding is permissible, and procedure for revealing a participant’s allocated intervention during the trial | N/A |
| **Methods: Data collection, management, and analysis** | | |  |
| Data collection methods | 25a | Plans for assessment and collection of trial data, including any related processes to promote data quality (e.g., duplicate measurements, training of assessors) and a description of trial instruments (e.g., questionnaires, laboratory tests) along with their reliability and validity, if known. Reference to where data collection forms can be accessed, if not in the protocol | 5-7 |
|  | 25b | Plans to promote participant retention and complete follow-up, including list of any outcome data to be collected for participants who discontinue or deviate from intervention protocols | 5 |
| Data management | 26 | Plans for data entry, coding, security, and storage, including any related processes to promote data quality (e.g., double data entry; range checks for data values). Reference to where details of data management procedures can be accessed, if not in the protocol | S2 |
| Statistical methods | 27a | Statistical methods used to compare groups for primary and secondary outcomes, including harms | 8, 9 |
|  | 27b | Definition of who will be included in each analysis (e.g., all randomized participants), and in which group | 8 |
|  | 27c | How missing data will be handled in the analysis | 8 |
|  | 27d | Methods for any additional analyses (e.g., subgroup and sensitivity analyses) | 8, 9 |
| **Methods: Monitoring** | | |  |
| Data monitoring committee | 28a | Composition of data monitoring committee (DMC); summary of its role and reporting structure; statement of whether it is independent from the sponsor and funder; conflicts of interest and reference to where further details about its charter can be found, if not in the protocol. Alternatively, an explanation of why a DMC is not needed | S2 |
|  | 28b | Explanation of any interim analyses and stopping guidelines, including who will have access to these interim results and make the final decision to terminate the trial | S2 |
| Trial monitoring | 29 | Frequency and procedures for monitoring trial conduct. If there is no monitoring, give explanation | 5, Figure 3, S2 |
| **Ethics** | | |  |
| Research ethics approval | 30 | Plans for seeking research ethics committee/institutional review board approval | 3, S2, S3, S17 |
| Protocol amendments | 31 | Plans for communicating important protocol modifications to relevant parties | S2, S3 |
| Consent or assent | 32a | Who will obtain informed consent or assent from potential trial participants or authorized proxies, and how | 3 |
|  | 32b | Additional consent provisions for collection and use of participant data and biological specimens in ancillary studies, if applicable | N/A |
| Confidentiality | 33 | How personal information about potential and enrolled participants will be collected, shared, and maintained in order to protect confidentiality before, during, and after the trial | 3, S2 |
| Ancillary and post-trial care | 34 | Provisions, if any, for ancillary and post-trial care, and for compensation to those who suffer harm from trial participation | N/A |

Supplemental Table 2. **WHO Trial Registration Data Set**

| **Data category** | **Information** |
| --- | --- |
| Primary registry and trial identifying number | ClinicalTrails.gov NCT06715358 |
| Date of registration in primary registry | 19 November, 2024 |
| Secondary identifying numbers | Regional Ethics Committee for Research with Medicinal Products (CEIm) of the Community of Madrid (CEIm-R) FSHE-SI-004 |
| Source(s) of monetary or material support | “la Caixa” Foundation (LCF/PR/CE16/10700001), SHE Foundation, and Fundación Nemesio Diez |
| Primary sponsor | Foundation for Science, Health and Education, Spain |
| Secondary sponsor(s) | “la Caixa” Foundation (LCF/PR/CE16/10700001) and Fundación Nemesio Diez |
| Contact for public queries | info@fundacionshe.org |
| Contact for scientific queries | estudio@fundacionshe.org |
| Public title | Cluster Randomized Trial for the SI! Program Reintervention for Elementary Schools |
| Scientific title | Rationale and design of a structured school-based reintervention of the SI! Program for child health promotion: a cluster-randomized controlled trial |
| Countries of recruitment | Spain |
| Health condition(s) or problem(s) studied | Cardiovascular health promotion in children |
| Intervention(s) | SI! Program Reintervention for Elementary: School-based intervention about diet, physical activity, body & heart and emotions with a reinforcement of the school environment. |
| Key inclusion and exclusion criteria | Inclusion criteria: children of 1^st^ grade of Elementary public schools and charter schools located in the Madrid Region or Mataró; having from 1st to 6th grades; at least one classroom in 1st grade; and a canteen. |
|  | Exclusion criteria: None |
| Study type | Interventional |
|  | Allocation: Cluster-randomized controlled trial |
|  | Primary purpose: Health promotion, prevention |
| Date of first enrollment | October 2023 |
| Sample size | 977 (Enrolled) |
| Recruitment status | Complete |
| Primary outcome(s) | 5-year changes from baseline in SI!-Child score (diet, physical activity, sleep health, and body mass index) |
| Key secondary outcomes | 5-year changes from baseline in SI!-Child subcomponents (diet, physical activity, sleep health, and body mass index), the Life’s Essential 8 score and subcomponents, sedentarism and adiposity (waist circumference, waist-to-height-ratio, and body fat) |
| Ethics Review | Approved (3 June, 2024) Regional Ethics Committee for Research with Medicinal Products of the Community of Madrid (CEIm-R)  +34 91 370 28 24  comite.regional@salud.madrid.org  C/ Aduana 2928013 Madrid |
| Completion date | 06-2031 (Estimated) |
| Summary results | - |
| IPD sharing statement | Yes (upon request) |

Supplemental Table 3. **Scoring criteria for the individual health metrics in the Life’s Essential 8 and the SI!-Child scores used in this study**

| **Individual metric** | **Life’s Essential 8** | **SI!-Child** |
| --- | --- | --- |
| **Diet** | MEDAS score**^*,^**^8^  100: 12–13 points 80: 10–11 points 50: 7–9 points 25: 4–6 points 0: 0–3 points | *Fruits & Vegetables:*  20: ≥5 s/day  15: 3–4 s/day  10: 1–2 s/day  0: <1 s/day  *Sugared beverages:*  20: <1 s/week  10: 1–3 s/week  5: 4–6 s/week  0: ≥ 7 s/week  *Fast-food:*  20 <1 s/week  10: 1 s/week  5: 2 s/week  0: ≥ 3 s/week  *Legumes & nuts:*  20: ≥ 10 s/week  15: 7–9 s/week  10: 4–6 s/week  0: 0–3 s/week  *Sweets & Pastries:*  20: <1 s/week  10: 1–3 s/week  5: 4-6 s/week  0: ≥ 7 s/week |
| **Physical Activity** *(MVPA minutes/week)* | 100: ≥420 min/week 90: 360–419 min/week 80: 300–359 min/week 60: 240–299 min/week 40: 120–239 min/week 20: 1–119 min/week 0: 0 min/week | 100: ≥420 min/week 90: 360–419 min/week 80: 300–359 min/week 60: 240–299 min/week 40: 120–239 min/week 20: 60–119 min/week 0: 0–59 min/week |
| **Nicotine exposure** *Combustible tobacco or NDS use (only in adolescents) or/and secondhand smoke exposure* | 100: Never tried tobacco 50: Tried tobacco 0: Current tobacco use  *Subtract 20 points (unless score is 0) for living with active indoor smoker in home* | 100: Never tried tobacco 50: Tried tobacco 0: Current tobacco use  *Subtract 20 points (unless score is 0) for living with active indoor smoker in home* |
| **Sleep health** *(Sleep hours/day)* | **Optimal range 6–12 y, 9–12 hours/day Optimal range 13–18 y, 8–10 hours/day**  100: Optimal range  90: <1 h above optimal range 70: <1 h below optimal range 40: 1–<2 h below or ≥1 h above optimal range 20: 2–<3 h below optimal range 0: ≥3 h below optimal range | **Optimal range 6–12 y, 9–12 hours/day Optimal range 13–18 y, 8–10 hours/day**  100: Optimal range 90: <1 h above optimal range 70: <1 h below optimal range 40: 1–<2 h below or ≥1 h above optimal range 20: 2–<3 h below optimal range 0: ≥3 h below optimal range |
| **Body mass index** | 100: <85^th^ percentile 70: 85^th^ –<95^th^ percentile 30: 95^th^ percentile–<120% of the 95^th^ percentile 15: 120% of the 95^th^ percentile–<140% of the 95^th^ percentile 0: ≥140% of the 95^th^ percentile | 100: 5^th^ –<85^th^ percentile 70: 85^th^–<95^th^ percentile and <5^th^ percentile 30: 95^th^ percentile–<120% of the 95^th^ percentile 15: 120% of the 95^th^ percentile–<140% of the 95^th^ percentile 0: ≥140% of the 95^th^ percentile |
| **Blood lipids** *(Non–HDL cholesterol (mg/dL))* | 100: <100 60: 100–119 40: 120–144 20: 145–189 0: ≥190  *Subtract 20 points if drug-treated level* | - |
| **Blood glucose** *(FBG (mg/dL))* | 100: No history of diabetes and FBG <100 60: No diabetes and FBG ≥100 40: Diabetes | - |
| **Blood pressure** *(Systolic and diastolic BP (mm Hg))* | **Age <13 years:**  100: Optimal (<90^th^ percentile) 75: Elevated (≥90^th^–<95^th^ percentile or ≥120/80 mm Hg to <95^th^ percentile, whichever is lower) 50: Stage 1 hypertension (≥95^th^–<95^th^ percentile+12 mm Hg, or 130/80 to 139/89 mm Hg, whichever is lower) 25: Stage 2 hypertension (≥95^th^ percentile+12 mm Hg, or ≥140/90 mm Hg, whichever is lower) 0: Systolic BP ≥160 or ≥95^th^ percentile+30 mm Hg systolic BP, whichever is lower; and/ or diastolic BP ≥100 or ≥95^th^ percentile+20 mm Hg diastolic BP  *Subtract 20 points if treated level*  **Age ≥13 years:**  100: <120/<80 (optimal) 75: 120–129/<80 (elevated) 50: 130–139 or 80–89 (stage 1 hypertension) 25: 140–159 or 90–99 0: ≥160 or ≥100  *Subtract 20 points if treated level* | - |

BP, Blood pressure. HDL, high-density lipoprotein. FBG, fasting blood glucose. MEDAS, Mediterranean Diet Adherence Screener. MVPA, moderate to vigorous physical activity. NDS, nicotine-delivery system. ^*^Excluding alcohol from the original score.

Supplemental Table 4. **Differences from baseline to each follow-up in the Life’s Essential 8 and the SI!-Child scores**

|  | **Life's Essential 8^*^**  **(mean change [95% CI])** | **SI!-Child^*^**  **(mean change [95% CI])** | **Between score difference^†^**  **(mean [95% CI])** | ***P* value** |
| --- | --- | --- | --- | --- |
| ***2-year follow-up*** *(n=547)* | –0.51 (–1.21 to 0.19) | –2.97 (-3.69 to –2.24) | –2.45 (–3.00 to –1.90) | **<**.**001** |
| ***4-year follow-up*** *(n=481)* | –2.56 (–3.31 to –1.80) | –7.82 (–8.72 to –6.92) | –5.19 (–5.80 to –4.58) | **<.001** |

^*^ Mean change from baseline to each follow-up for each score. **^†^** Difference in mean change from baseline between the two scores at each follow-up. Statistical comparisons were made using unpaired t-tests.

# SUPPLEMENTAL FIGURES

**
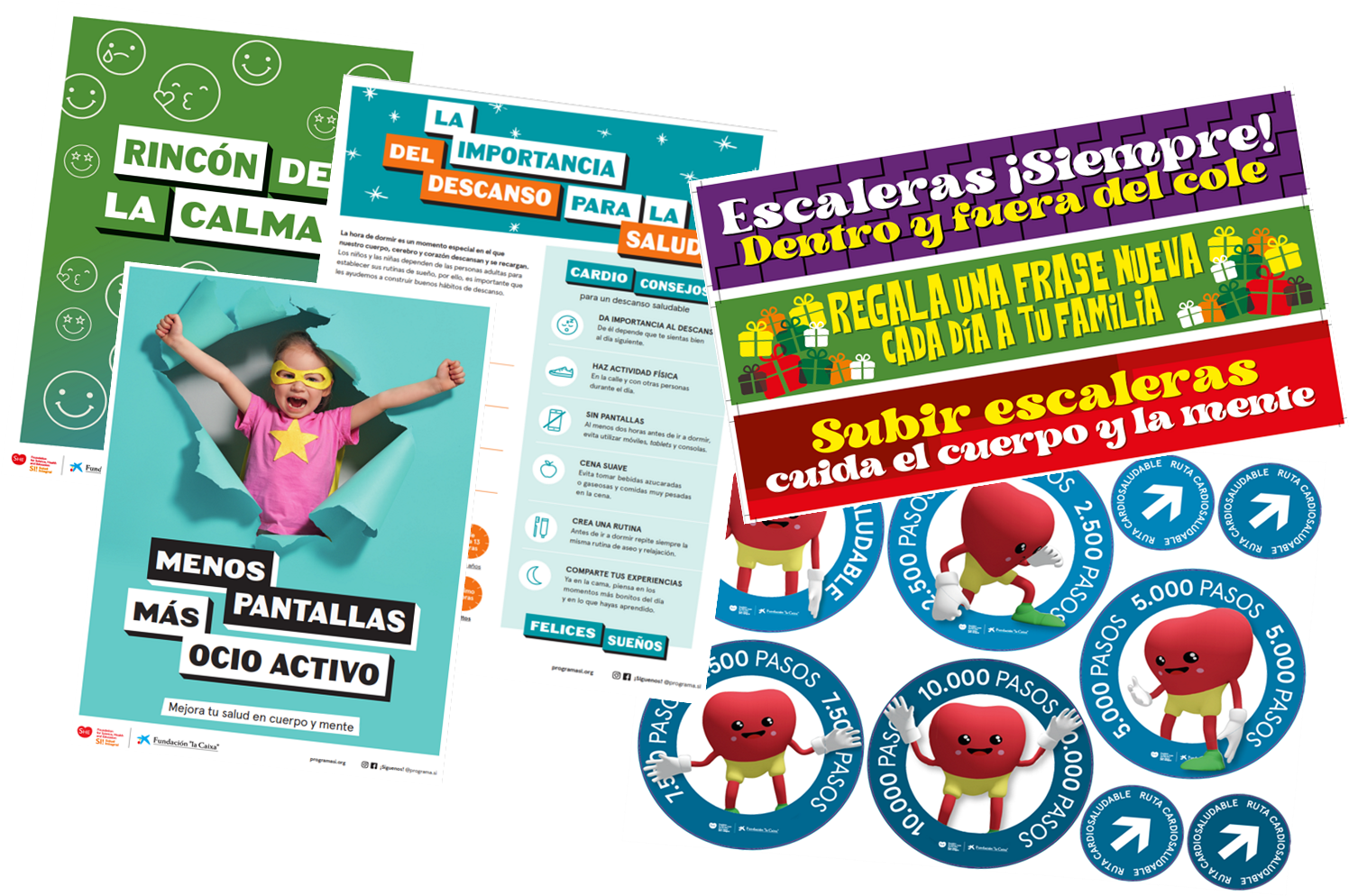
**Supplemental Figure 1**. Sample welcome pack for schools implementing the SI! Program**

Supplemental Figure 2. **Healthmeter interactive tool in the SI! Program**


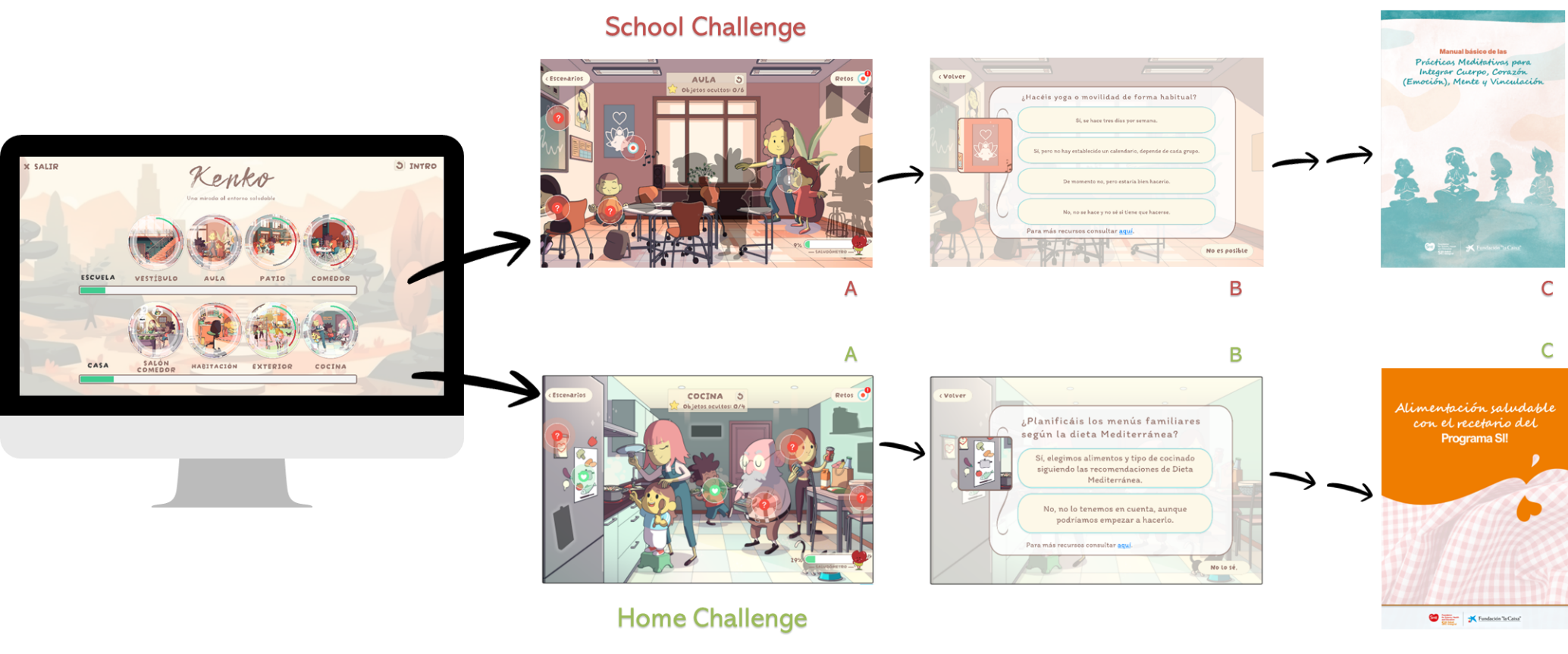


A: Guidance to help users identify and implement improvements in school and household routines; B: Example of questions presented during navigation; C: Example of an educational resource provided by the platform to support improvements in the assessed area.

Supplemental Figure 3. **Classification of cardiovascular health according to the Life’s Essential 8 and SI!-Child scores**


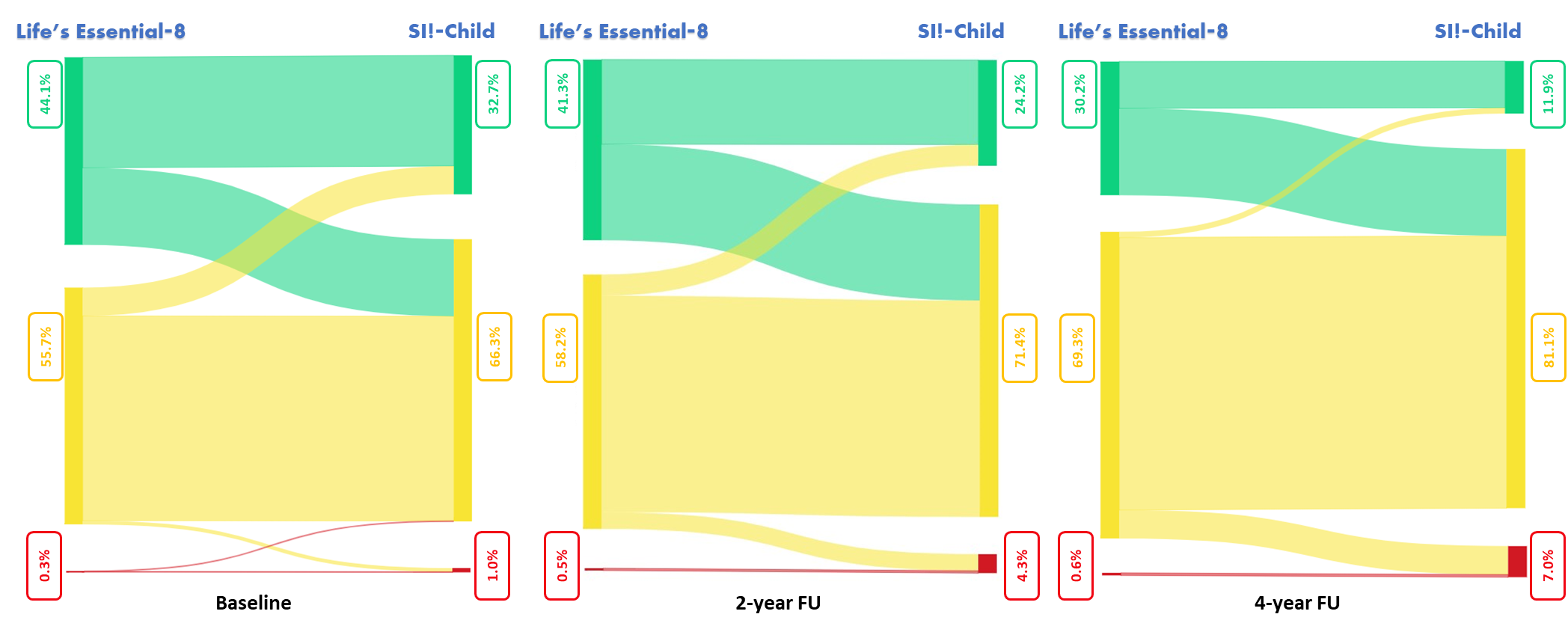


Overall scores on the Life’s Essential 8 and SI!-Child scores were categorized as poor (0–49 points, red), intermediate (50–79 points, yellow), or ideal (80–100 points, green). FU, follow-up

Supplemental Figure 4. **SI!-Child score vs. Life’s Essential 8: between-score agreement for overall lifestyle-related metrics**


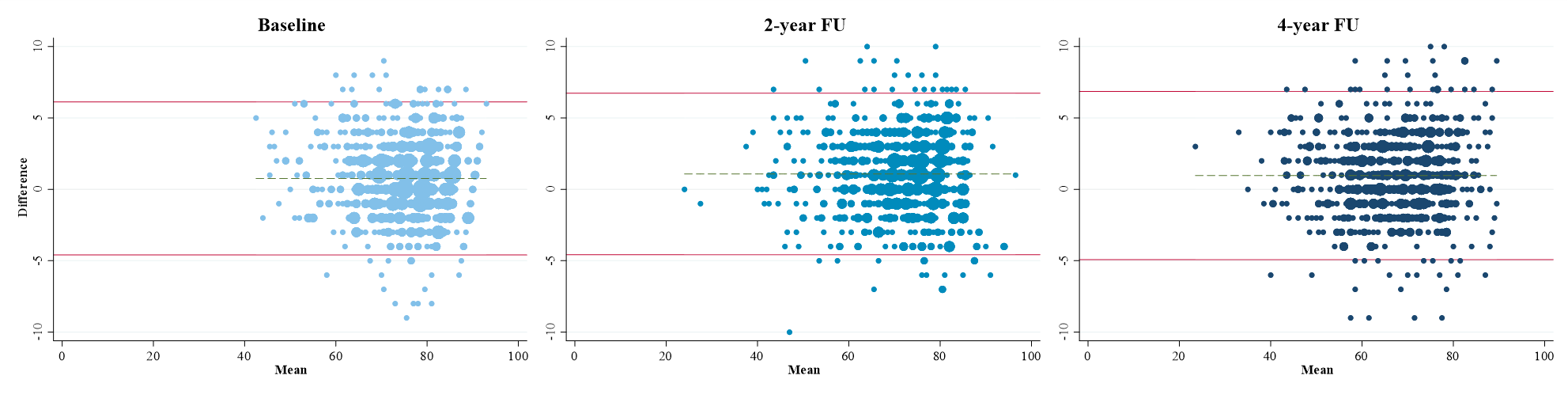


Bland–Altman plots showing agreement between the SI!-Child and Life’s Essential 8 scores at baseline and at 2- and 4-year follow-up. Discontinuous green lines represent the mean difference, and red lines represent limits of agreement. FU, follow-up.

Supplemental Figure 5**. SI!-Child score vs. Life’s Essential 8: between-score agreement for individual lifestyle-related metrics**


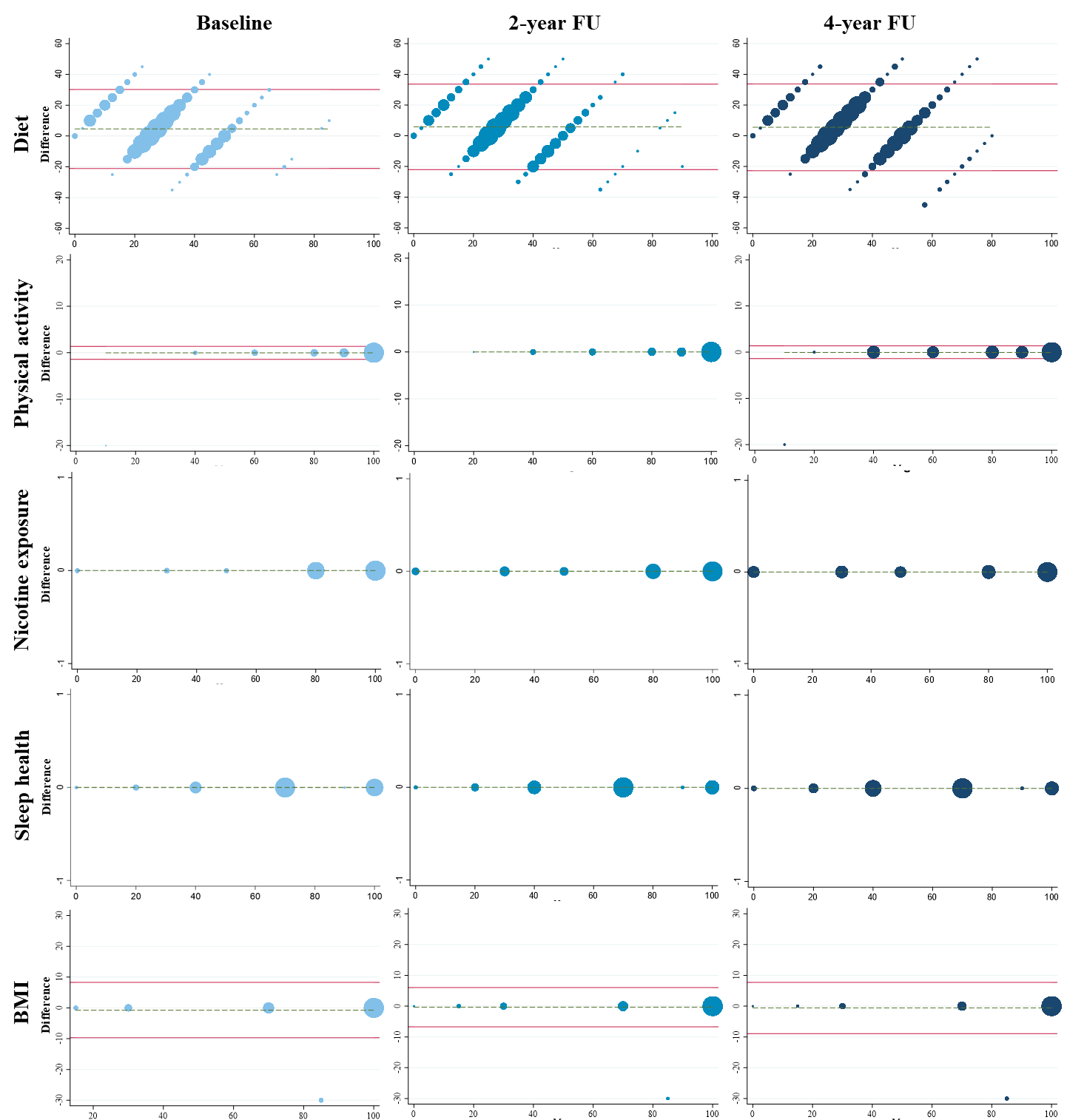


Bland–Altman plots showing agreement between the SI!-Child and Life’s Essential 8 scores across individual lifestyle-related metrics. Discontinuous green lines represent the mean difference, and red lines represent limits of agreement. BMI, body mass index; FU, follow-up.

# SUPPLEMENTAL REFERENCES

1. Fernandez-Jimenez R, Santos-Beneit G, Tresserra-Rimbau A, et al. Rationale and design of the school-based SI! Program to face obesity and promote health among Spanish adolescents: A cluster-randomized controlled trial. *Am Heart J*. 2019;215:27-40. doi:10.1016/j.ahj.2019.03.014

2. Santos-Beneit G, Fernández-Alvira JM, Tresserra-Rimbau A, et al. School-Based Cardiovascular Health Promotion in Adolescents: A Cluster Randomized Clinical Trial. *JAMA Cardiol*. 2023;8(9):816-824. doi:10.1001/jamacardio.2023.2231

3. Lloyd-Jones DM, Allen NB, Anderson CAM, et al. Life's Essential 8: Updating and Enhancing the American Heart Association's Construct of Cardiovascular Health: A Presidential Advisory From the American Heart Association. *Circulation*. 2022;146(5):e18-e43. doi:10.1161/CIR.0000000000001078

4. Appel LJ, Moore TJ, Obarzanek E, et al. A clinical trial of the effects of dietary patterns on blood pressure. DASH Collaborative Research Group. *N Engl J Med*. 1997;336(16):1117-24. doi:10.1056/NEJM199704173361601

5. Cerwinske LA, Rasmussen HE, Lipson S, Volgman AS, Tangney CC. Evaluation of a dietary screener: the Mediterranean Eating Pattern for Americans tool. *J Hum Nutr Diet*. 2017;30(5):596-603. doi:10.1111/jhn.12451

6. Gil A, Martinez de Victoria E, Olza J. Indicators for the evaluation of diet quality. *Nutr Hosp*. 2015;31 Suppl 3:128-44. doi:10.3305/nh.2015.31.sup3.8761

7. Grao-Cruces A, Velasquez-Romero MJ, Rodriguez-Rodriguez F. Levels of Physical Activity during School Hours in Children and Adolescents: A Systematic Review. *Int J Environ Res Public Health*. 2020;17(13)doi:10.3390/ijerph17134773

8. Martinez-Gonzalez MA, Garcia-Arellano A, Toledo E, et al. A 14-item Mediterranean diet assessment tool and obesity indexes among high-risk subjects: the PREDIMED trial. *PLoS One*. 2012;7(8):e43134. doi:10.1371/journal.pone.0043134
